# Increased chemical weathering during the deglacial to mid-Holocene summer monsoon intensification

**DOI:** 10.1038/srep44310

**Published:** 2017-03-17

**Authors:** Pavan Miriyala, N. P. Sukumaran, B. Nagender Nath, P. B. Ramamurty, A. V. Sijinkumar, B. Vijayagopal, V. Ramaswamy, Tyson Sebastian

**Affiliations:** 1 Geochronology & Isotope Studies, CSIR-National Geophysical Research Institute, Hyderabad, India; 2 Geological Oceanographic Division, CSIR-National Institute of Oceanography, Goa, India; 3 Department of Geology, Central University of Kerala, Kerala, India

## Abstract

Chemical weathering and the ensuing atmospheric carbon dioxide consumption has long been considered to work on geological time periods until recently when some modelling and natural records have shown that the weathering-related CO_2_ consumption can change at century to glacial-interglacial time scale. Last glacial to interglacial transition period is a best test case to understand the interplay between Pco_2_-temperature-chemical weathering when a pulse of rapid chemical weathering was initiated. Here we show, from a high resolution 54 ka record from the Andaman Sea in the northern Indian Ocean, that the chemical weathering responds to deglacial to mid-Holocene summer monsoon intensification in the Myanmar watersheds. The multi-proxy data (Al/K, CIA, Rb/Sr, ^87^Sr/^86^Sr for degree of weathering and ^143^Nd/^144^Nd for provenance) reveal an increase in silicate weathering with initiation of interglacial warm climate at ~17.7 ka followed by a major change at 15.5 ka. Inferred changes in chemical weathering have varied in tandem with the regional monsoonal proxies (δ^18^O_sw_-salinity changes of Northern Indian Ocean, effective Asian moisture content and δ^18^O records of Chinese caves) and are synchronous with changes in summer insolation at 30°N and δ^18^O of GISP2 implying that chemical weathering was not a later amplifier but worked in tandem with global climate change.

Silicate weathering is the major process which consumes Pco_2_ and preserves its imprints in the weathered products. While the weathering controlled solute transport in the present day streams/water bodies fail to detect the climatic link^1^, weathering rates in soils and small catchments were found to be linked to significant climate effect^2^. Consequently, the role of chemical weathering has been considered only in studies dealing with geological climatic evolution, because it was thought to react slowly to climatic changes. A recent report, of a rapid increase in the chemical weathering flux in Iceland (up to 30% increase over four decades) in response to high-latitude climate change^3^, however has challenged this idea. More recent modeling study of MacKenzie River basin, an Arctic watershed has supported this. At the current rate of anthropogenic emissions and increased atmospheric CO_2_ and temperature, it was estimated that the CO_2_ consumption flux related to weathering processes increases by more than 50% for an atmospheric CO_2_ doubling by the end of this century^4^. In this context, paleoweathering records of climate change regime would be useful to assess their linkage. Major climatic changes occurred during the glacial-interglacial cycles during the Quaternary. The current interglacial has also witnessed short term but profound climatic changes such as Bolling-Allerod (B-A), Younger Dryas (YD) and Little Ice Age. Lab experiments by Berner (BLAG)^5^, Walker (WAGH)^6^ Volk^7^ and others^1^ concluded “temperature as the driving force” based on rates of mineral dissolution by changing temperature and pH of fluids. Whether the climatic changes involving atmospheric temperature have triggered the erosion and weathering in the tropical areas is crucial in our assessment of climate-CO_2_-weathering linkages. Monsoon is the important climatic phenomenon in Asia which involves temperature and humidity changes both critical for silicate weathering. Here, we present a multi-proxy-based ~54 ka weathering record from the Alcock seamount complex in central Andaman Sea (Fig. 1 - SK168/GC1 hereafter referred as SK168), Northern Indian Ocean in the context of this debate (Supplementary, Materials and Methods).

Andaman basin is particularly suitable for such studies as it is a confined basin, separated from the Bay of Bengal by islands and sills, the contributing terrigenous sources^8^ and the oceanography are well constrained^9^,^10^. The terrigenous sediments are principally derived from Myanmar rivers (Irrawaddy, Salween and Sittang) which combinedly supply more than 528 million tons of sediments annually, and are funneled through Gulf of Martaban to deep Andaman basin^11^. A single record of weathering is available for this area which covers a long period (~280 ka^12^) but with a low resolution (~8 data points covering the time period presented here) which preludes the study of weathering changes in response to multiple climatic events occurred since the Last Glacial Maximum (LGM). Past salinity and paleoceanographic variations were already elucidated^13^ for the new weathering record presented here.

## Results and Interpretations

Grain size and geochemistry of lithogenic fraction of core SK 168 and Myanmar shelf sediments evaluates the changes in sediment provenance with varying time. The median grain size varied narrowly between 3 and 7μm (Fig. 2) and hence the strontium isotope variation is not influenced by size fractionation. Median size displayed 3 periods (see Fig. 2 for 3-point moving average) of larger grain size at, 1) 36-33 ka, 2) 23-19 ka and 3) 15.5-7 ka. While the 36-33 ka and 15.5-7 ka peaks of coarsening coincide with 2 major peaks of solar insolation (Fig. 2), the 23-19 ka period marks the peak glacial time and probably reflects the intensification of physical weathering. As shown in later part of the paper, 15-7 ka is the period of increased monsoon (and runoff) and also associated with intensified chemical weathering. The shale normalized La to Yb ratio^14^ (Fig. 2), has varied between 0.9 and 1.3 suggesting that the overall source has fluctuated between felsic to intermediate composition. The ratio shows a gradual decrease from the end of the record to LGM (La_n_/Yb_n_ ~1.3 to 1.1) and acquires a near-flat shale-normalized pattern and indicates that the sediment composition has either shifted from a dominantly felsic source to intermediate rock sources^15^ or may suggest an efficient mixing in the drainage areas since the deglaciation. Between LGM to present day, the ratio fluctuates between 1 and 1.2 reflecting periods of minor changes in provenance or changing efficiency in sediment mixing. The major and trace element data of sediments from Myanmar continental shelf and SK168 are plotted in the geochemical discrimination plots (Fig. S3). All the samples from SK168 cluster closely, while the shelf samples fall at the edges of SK168 cluster (Fig. S3a), probably as a result of well mixing of Myanmar river materials. A slight shift in shelf values away from the SK168 in felsic-mafic ternary plot (Fig. S3b) might be due to higher Al content in shelf sediments due to its close proximity to the Myanmar river mouths.

The Sm-Nd depleted model age^16^ T_DM_ calculated for SK168 core varies between 1.14 to 1.46 Ga and the average remained close to 1.33 Ga for both glacial and interglacial sediments, which suggests that the provenance remained same through the climatic change. The T_DM_ of shelf sediments (1.3 to 1.4 Ga) fall very close to SK168 values and thus the sediment deposited in Alcock area was mainly carried by Myanmar Rivers.

### Interpreting the provenance

Nd isotopic compositions of the shelf sediments off river mouths Irrawaddy, Salween, Sittang and Arakan coast track the present day pathways of terrigenous sediment transport to the deep Andaman Sea. The more radiogenic ε_Nd_ in Irrawaddy mouth shelf sample SK175/38 (ε_Nd_ −8.72 ± 0.33) is very similar to the Arakan shelf sample SK175/3 (ε_Nd_ −8.88 ± 0.33), and agrees well with reported Irrawaddy sediment value of ε_Nd_ −8.3^17^ and implies that the eastern and western part of Indo-Burman ranges (IBR) are contributing near similar Nd signal to the open ocean, either through Arakan, Rakhine rivers in western IBR or through Irrawaddy river on eastern IBR. On the other hand, shelf sediments off rivers Salween (SK175/52; ε_Nd_ −10.37 ± 0.33) and Sittang (SK175/48; ε_Nd_ −11.19 ± 0.33) have distinctly low radiogenic values reflecting a dominance of old crustal material in the flood plains of Salween and Sittang. The river Salween originates in eastern syntaxes of Himalaya and flows through south-eastern Tethyan tectonic belt^18^,^19^, while river Sittang originates and flows entirely from Shan Plateau of Sino-Burman ranges (SBR). The clockwise movement of stronger monsoon currents probably mix the Irrawaddy, Salween and Sittang sediments well during the transport^11^,^20^ and deposition in the Central Andaman basin.

**Figure 1:**
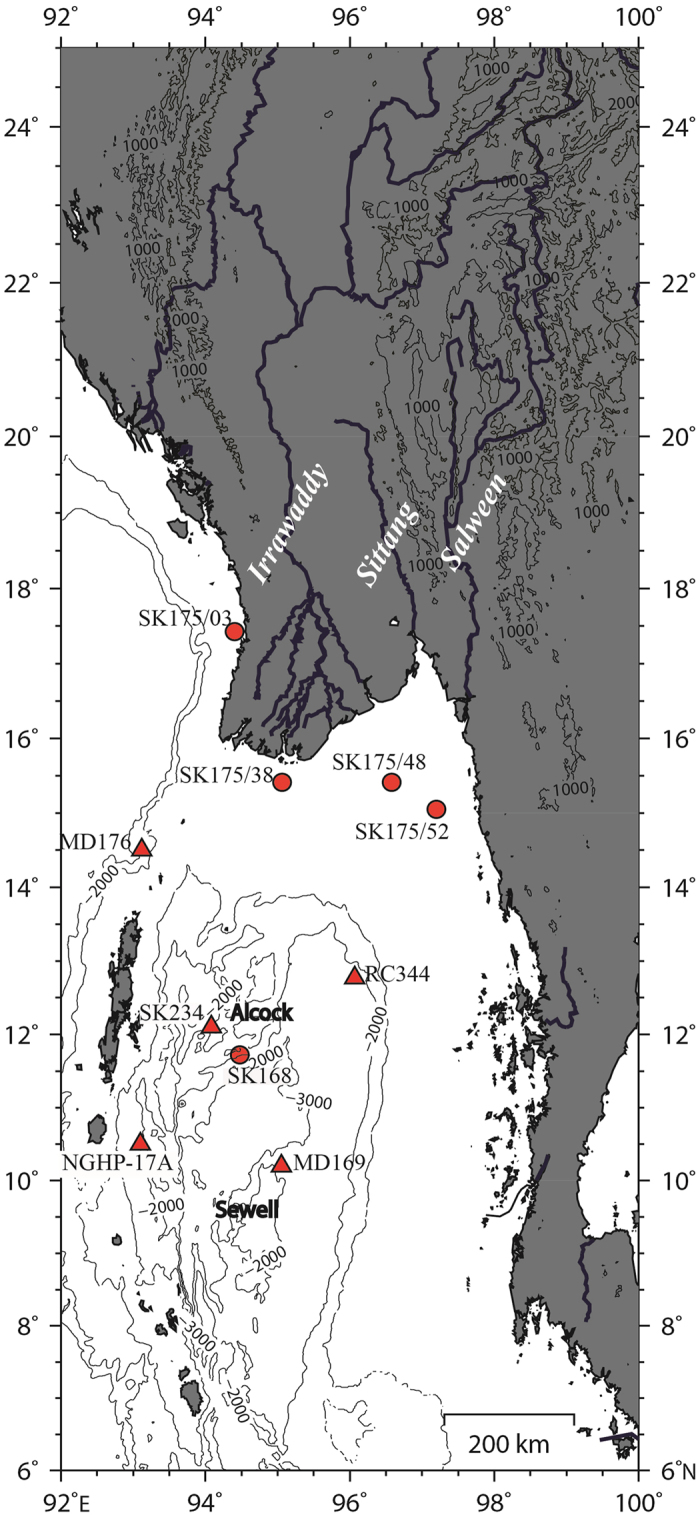
Location map (generated by using GMT^68^ (v4.5)) of (**a**) elevation contours and river paths of Myanmar, bathymetry of the Andaman Sea along with; (**b**) Alcock and Sewell seamount complex; (**c**) shelf sediments of Arakan (SK175/3), river mouths off Irrawaddy (SK175/38), Sittang (SK175/48), Salween (SK175/52), gravity core SK168 (all from this study); (**d**) published records MD77-169, MD77-176, RC12-344^12^; SK234/60^22^; NGHP01-17A^23^.

The isotopic data of core SK168 (this work - northern half of the Central Andaman Trough - CAT) exhibits a narrow range of Nd isotopes (ε_Nd_ −11.24 to −9.24; average −10.15 ± 0.46) (Fig. 3) which fall very close to other published CAT values (MD77-169^12^ of Sewell seamount (here after MD169) and RC12-344^12^ (hereafter RC344)), (ε_Nd_ −11.5 to −9.5; average −10.56 ± 0.3) implies the CAT region of Andaman Sea had received material from a same source during the last two glacial cycles. Another record MD77-176 (hereafter MD176) located close to Irrawaddy mouth shows high radiogenic Nd^12^ of −9.2 to −7.7 with average value of −8.67 ± 0.43 (Fig. 3b). High radiogenic ε_Nd_ in MD176 is similar to Arakan shelf (SK175/3 - this study) and Irrawaddy mouth (SK175/38 - this study) sediments. All these 3 areas seem to reflect the dominant supply from Irrawaddy; whereas the CAT cores received low radiogenic Nd material from Salween and Sittang rivers. The slight enrichment of unradiogenic material in glacial sediments of CAT cores may be due to climatic change (intensified NE monsoon and weakened SW monsoon in the Myanmar plains). The possibility of addition of material from Andaman islands to the CAT is ruled out due to absence of major rivers on islands and the presence of coral reefs which grew since LGM^21^. Two other records from the western part of Andaman Sea^22^,^23^ (Fig. 1) have reported high radiogenic Nd. The range of ε_Nd_ (−7.3 to −5.3) and the average value of −6.04 ± 0.53 (Fig. 3b) of core NGHP-17A^23^ lies between beach sediment values of middle Andaman (ε_Nd_ −5.1 ± 0.50)^23^ and Neil islands (ε_Nd_ −7.6 ± 0.50)^23^ suggesting a major contribution from the local sources to the western AS. In an another core SK234^22^ collected near Barren volcano, ε_Nd_ vary between −9.0 and −5.3 (average −7.4 ± 1.1) (Fig. 3b) and ^87^Sr/^86^Sr 0.70861 to 0.71680 (Fig. 3a). The major portion of SK234 record has high radiogenic ε_Nd_ > −8.0 and less radiogenic Sr < 0.712. This core has multiple ash layers^24^ (5.75 ± 0.54) derived from Barren volcanism and less likely to receive terrigenous sediments from the Myanmar rivers^8^. As shown above, when the average values of ε_Nd_ in the Andaman sediment are compared, a regional variation in the source provenance of the detrital sediments of the Andaman Sea can be constructed. The CAT region is depleted by ~2 ε_Nd_ units relative to Irrawaddy mouth region, and ~4 ε_Nd_ units relative to the western region (NGHP-17A and SK234). The observed high radiogenic Nd of NGHP-17A core and the large excursions in isotopic ratios of SK234 (Barren) are not seen in the CAT cores and suggest that the central Andaman Sea has little supply from island and the volcanic sources in the western AS (Fig. S4). The close variation in glacial ε_Nd_ of SK168 with Salween and Sittang river mouth sediments and interglacial ε_Nd_ with Irrawaddy river and Arakan coast suggest that the CAT region is mainly fed by the Irrawaddy-Salween-Sittang (ISS) river systems. Thus, the CAT is the most suitable location to explore the climatic impacts on Myanmar continental region.

The ^87^Sr/^86^Sr ratio in core SK168 varies from 0.715421 to 0.724377 (Fig. 3a) which distinctly vary at different climatic regimes, with higher ratios in SK168 and other CAT cores during glacial and lower during humid interglacial periods implying reduced chemical weathering during glacial times and vice-versa during interglacials (Fig. 3a). The weighted average ε_Nd_ values for the time slices representing glacial conditions (54–17.7 ka), the last glacial - interglacial transition (hereafter deglacial) (18–11.6 ka), and the interglacial starting at 11.6 ka are −10.43 ± 0.20 (n = 25), −10 ± 1.74 (n = 6) and −9.69 ± 0.38 (n = 15) in SK 168 nearly brackets the range of values that we observe in the river mouth samples of Irrawaddy-Salween-Sittang (ISS) river systems.

A simple mixing calculation assuming two end members, of a less radiogenic end-member representing the Salween and Sittang rivers (weighted average ε_Nd_ −10.79) and a more radiogenic end member representing the river Irrawaddy and Arakan coast (weighted average ε_Nd_ −8.82), shows that during the glacial period, nearly 82% of the material came from the Salween and Sittang rivers and only 18% from the river Irrawaddy. For the deglacial, 60% of the detritus were sourced from Salween and Sittang rivers as against 40% from Irrawaddy. For the present interglacial, the Irrawaddy dominates the Salween and Sittang rivers by 56%. More importantly, the period between 15.5 and 5.5 ka, the main focus of this study, the Irrawaddy contributes more than 60% of the detritus material to our site. This calculation assumes that the river mouth samples represent the average composition of the catchment origin from which the rivers derive their loads. The new high resolution data of Nd isotopes of SK168 in combination with published CAT (MD169 and MD176)^12^ records has helped in new interpretations. A shift towards high radiogenic Nd values is observed between 15.5 and 5.5 ka in SK168, which is not clearly visible in MD169 & MD176 due to low resolution but a kink of high radiogenic Nd at 9.8 ka^12^ is synchronous in all records (Fig. 3b), implying a similar forcing mechanism that has taken place in the source area during the weathering. Although the CAT records show clear glacial-interglacial variability in ε_Nd_ or ^87^Sr/^86^Sr, no discernible changes are apparent during the North Atlantic climatic events of YD and B-A.

## Discussion

Temporal changes in chemical weathering intensity (interpreted from proxies Al/K, Rb/Sr, CIA^25^ and ^87^Sr/^86^Sr – Fig. 4) mark three broad time periods in this 54 ka record, the glacial (54 to 17.7 ka), the deglacial (17.7 to 11.6 ka) and interglacial periods (11.6 ka to present day). The overall CIA* (defined in Fig. 4) ranges from 80 to 83, implies moderate to high chemical weathering in the source regions. The average Al/K ratios are low (4.2) in glacial, high (4.8) in interglacial with intermediate values (4.6) during deglacial since LGM reflecting the highest degree of chemical weathering during interglacial period. Rb/Sr ratios on an average are low during interglacials (1.2) and during deglacial (1.4) than glacials (1.7), the reduction of Rb with an increase in Sr as an effect of enhanced chemical weathering. The same is reflected with low ^87^Sr/^86^Sr ratios during interglacial (0.71882 ± 0.007 n = 15) and deglacial (0.71994 ± 0.007 n = 6) and higher ratios (0.72238 ± 0.007 n = 25) in glacial sediments (Fig. 3a). Higher ^87^Sr/^86^Sr ratios during glacials shows the dominance of physical weathering. Least radiogenic value (0.71542) is noticed at 17.7 ka, the transition to warmer climate and increased chemical weathering. Chemical weathering changes were not evident during YD probably responding more to local summer insolation and/or due to insufficient sediment residence time for the chemical weathering to take place. Low radiogenic Sr during 9-7 ka of Holocene implies increased chemical weathering, consistent with the timings of increased monsoon induced erosion in the western Himalayas^26^. Interestingly the highest effective moisture is recorded at same time (9-7 ka) in Asian moisture (Fig. 4), and strongly suggest that 9-7 ka is an intensified monsoonal event in south Asian climate.

The reduced salinity from 36 to 31psu between 16 and 4 ka in a record from Ganges river mouth (126KL) of Bay of Bengal^27^, shows that intensification of summer monsoon with initiation of interglacial climate. This increased fresh water input is reflected in δ^18^O_sw_ records of western BoB (SK218)^28^ and the Andaman Sea (RC344)^29^, and shows that the entire Northeastern Indian Ocean received fresh water during this intensified SW monsoon^13^. These monsoonal records are synchronous to summer insolation at 30°N^30^ implying the role of solar insolation in enhancing SW monsoon.

**Figure 2:**
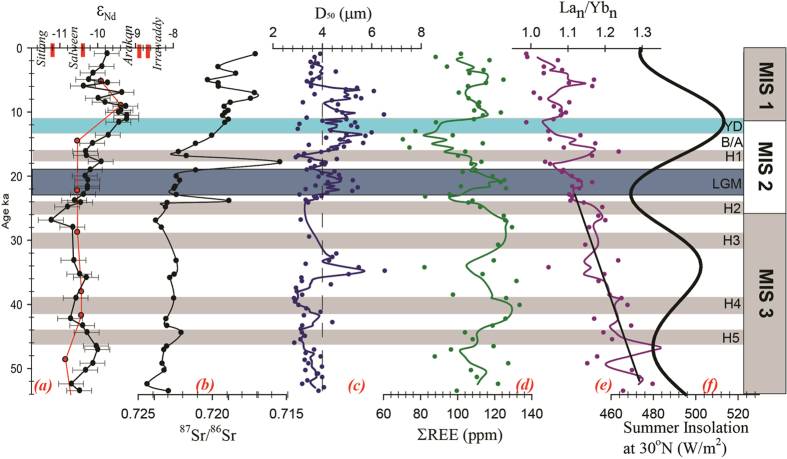
Downcore variations of (**a**) ε_Nd_ of SK168 of Alcock seamount (this study - with 2sd error bars) and MD169 of Sewell seamount^12^ in the Central Andaman Trough (CAT); (**b**) ^87^Sr/^86^Sr of SK168 (this study); the 2sd errors of Sr isotopes are smaller than the symbol size; (**c**) 3-point moving average median grain size (D_50_), and dotted vertical line indicates average D_50_ for entire core; (**d**) 3-point moving average concentration of total rare earth elements; (**e**) 3-point moving average PAAS normalized ratios of light (La) to heavy (Yb) REE^14^; trend line for glacial period showing a reduction from ~1.3 to 1.1; (**f**) summer insolation at 30°N^30^. Shaded regions denote the major climatic events (YD-Younger Dryas, B/A-Bolling/Allerod, H1-H5- Heinrich events and LGM-Last Glacial Maximum). MIS1-3 indicate marine isotope stages.

**Figure 3:**
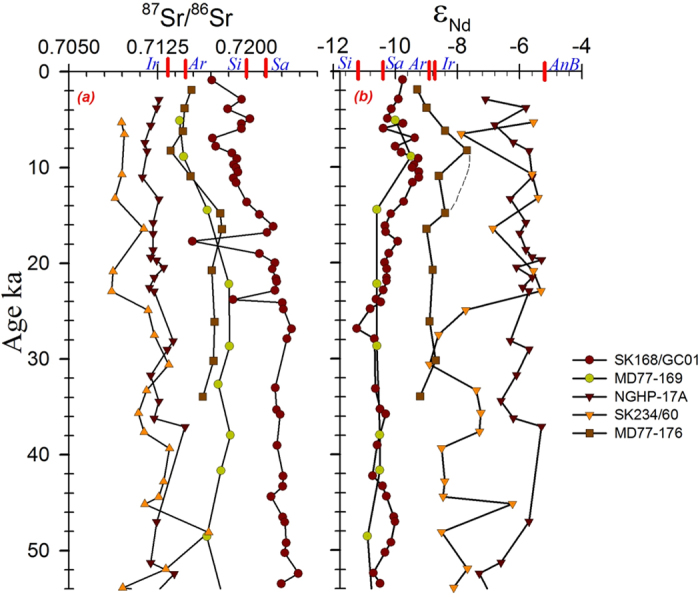
All available (**a**) ^87^Sr/^86^Sr and (**b**) ε_Nd_ records in and around Andaman Sea along with new Alcock seamount core data (SK 168 - this study). Dashed line in ε_Nd_ of MD176 indicates proposed increase in radiogenic nature observed in central Andaman Sea cores. The red markings on x-axis indicate the isotopic values of sediments from continental shelf off river mouths and Middle Andaman island beach (names abbreviated). Ar-Arakan coast, Ir-off Irrawaddy, Sa-off Salween, Si-off Sittang and AnB-Andaman island beach sediment.

**Figure 4:**
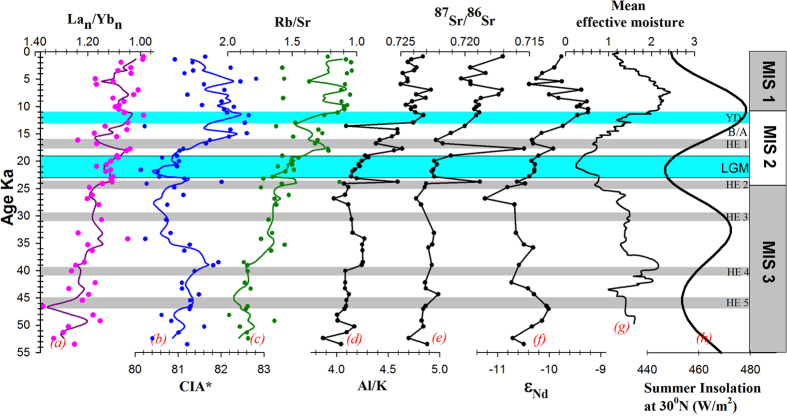
Downcore plots of SK168 (present study); (**a**) La_n_/Yb_n_ ratio and 3 point moving average; (**b**) Chemical Index of Alteration* and 3 point moving average (CIA*-excluding CaO^12^) calculated from molar proportions of major elements; (**c**) elemental ratio of rubidium to strontium and its 3-point moving average; (**d**) elemental ratio of aluminium to potassium; (**e**) ^87^Sr/^86^Sr; (**f**) ε_Nd_; (**g**) mean effective moisture of Asia^36^; (**h**) summer insolation at 30°N^31^. La_n_/Yb_n_, Rb/Sr and Sr/Sr are in reverse order to get best fit with other plots. Descriptions for shaded region can be found in Fig. 2 caption.

The SW monsoon has weakened and NE monsoon has strengthened during glacials^31^,^32^. The impact of southward shifting of the locus of Intertropical Convergent Zone (ITCZ) and increased rainfall in rivers of southern Arakan coast were found to have contributed LGM sediments with high radiogenic Nd to Bay of Bengal^33^,^34^. The lack of such radiogenic Nd in the glacial CAT (both in Alcock (this study) and the Sewell seamount record^12^) suggests that the rivers flowing through SBR (Salween and Sittang) may be the major contributors to the CAT. Absence of such radiogenic Nd shifts in these CAT cores suggests the dominance of NE monsoon during glacial times, which ultimately brought unradiogenic Nd from river basins of Salween and Sittang of SBR. During glacials, due to the sea level fall and exposure of shelf, eastward flowing currents would have weakened and isolated Irrawaddy from Salween and Sittang sources (Fig. S5). The time series plot of ε_Nd_ displays two humps of radiogenic nature during 53 to 42 ka and 15.5 to 5.5 ka periods (Fig. 2). The first radiogenic event at 53 to 42 ka occurred during low summer insolation, coincident with Heinrich event 5 recognized in North Atlantic Ocean. There was no change in ε_Nd_ during LGM at 23 to 19 ka^35^ as the source remained constant in this peak of arid climate. High radiogenic ε_Nd_ (−9.92) and a peak in all chemical weathering proxies (Fig. 4) with the occurrence of first warmth event in effective moisture content of Asian atmosphere^36^ at 17.7 ka marks this period as the first trigger of warm climate. But it took more than 2 thousand years to show a clear impact of warm climate due to coverage of ice sheets formed during glacials. The significant shift in radiogenic nature of Nd at 15.5 ka may mark the beginning of warm period and increased monsoon which continued upto 5.5 ka. This increased radiogenic Nd input is also observed in other two Myanmar river fed low resolution records of Sewell seamount (MD169) and Irrawaddy river mouth (MD176)^12^ during this period of SW monsoonal intensification^29^. Interestingly, changes in all chemical weathering proxies Al/K, Rb/Sr, ^87^Sr/^86^Sr co-vary with this high radiogenic Nd event and establishes a link between enhanced chemical weathering and supply of radiogenic Nd to deep Andaman Sea.

The present day rainfall data during 1988–1997 (Department of Meteorology and Hydrology of Myanmar) shows the dominance of SW monsoon (~92%) over NE monsoon on the plains of Myanmar Rivers. As major part of Central Myanmar Basin has a dry zone (CDZ) formed due to rain shadow of Rakhine mountains, the sediment loads of Irrawaddy will have much influence of IBR flux^37^,^38^. CDZ receives very low rainfall ~600 mm, while the northern and southern part of CDZ receives around 2300 and 1200 mm of rainfall respectively. But, these are much lower than IBR located on western side of Irrawaddy basin (Rakhine, Chin and Arakan hills) which receives about 5050 mm. Thus IBR may have a larger control on sediment of Irrawaddy that is carried to the deep sea.

The Mg/Ca values of RC344 core shows the Sea Surface Temperature (SST) during early Holocene (27.9 °C) is closer to modern August SST (28.6 °C)^29^. Thus if one considers the present day rainfall (5050 mm) is similar to that during the deglacial to mid Holocene, it is possible that IBR catchment could be the potential source for the weathered materials to Irrawaddy river.

**Figure 5:**
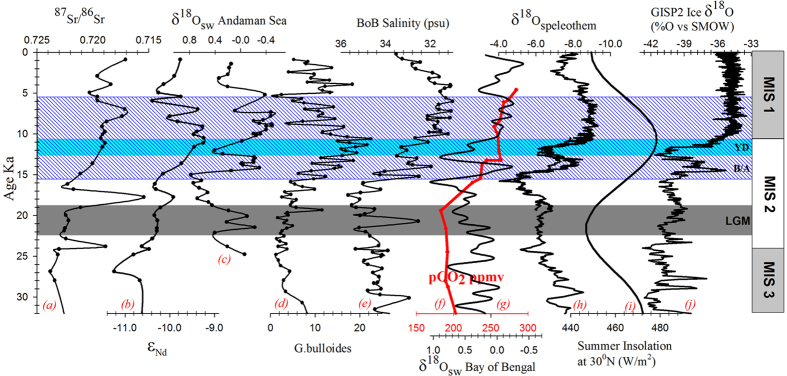
Downcore plots of (**a**) ^87^Sr/^86^Sr of SK168 (present study); (**b**) εNd of SK168 (present study); (**c**) δ^18^O_sw_ of Andaman Sea of RC12-344^29^; (**d**) Planktonic foraminifer (Globigerina Bulloides) % in SK168^35^; (**e**) Salinity record in 126KL core of Bay of Bengal^27^; (**f**) pCO_2_^39^; (**g**) δ^18^O_sw_ of middle-west Bay of Bengal from SK218^28^; (**h**) δ^18^O of stalagmites of Chinese caves^69^,^70^; (**i**) summer insolation at 30°N^31^; (**j**) δ^18^O of ice core from Greenland ice-sheet program GISP2^71^. For clarity we have restricted the time series records to 32 ka. Descriptions for shaded region except blue can be found in Fig. 2 caption. The blue color shaded region represents the period with coeval increase in chemical weathering and the deglacial monsoon.

The interglacial climate is known for high humidity, large day-night temperature variations, high monsoonal precipitation, together leading to increase in vegetation cover, soil zone and rain water residence time which is more acidic due to increased atmospheric CO_2_^39^ content (Fig. 5). All these would favor an increased chemical weathering conditions at the source region.

Atmospheric CO_2_ concentrations in the Northern Hemisphere is said to lead the global temperature making it an important driver of deglacial warming^40^. With the rise in atmospheric PCO_2_ concentrations, the deglacial warming initiated at 17 ka is coeval with the intensification of chemical weathering in Myanmar watersheds (Fig. 5). This implies that the atmospheric CO_2_ rise was a trigger for monsoon, which in turn had intensified the silicate weathering in the source regions. A part of risen deglacial CO_2_ would have been consumed during the deglacial to mid-Holocene weathering.

Given that the basic and ultrabasic materials tend to weather more quickly than the high crystalline rocks because of textural differences^41^, the IBR region which is characteristic of recent volcanic dykes, ophiolites, mud volcanoes, flysch^17^,^22^,^42^,^43^,^44^ could have weathered preferentially. It is probable that the increased catchments due to intensified monsoon might have energized the supply of radiogenic Nd to the total sediment discharge of Irrawaddy River.

A 54 ka lithogenic sediment depositional record from the Andaman Sea tracked monsoon variations, weathering patterns, and provenance changes with time. Climate-driven changes in chemical weathering and erosion in the Myanmar river catchments on these time scales is evident. More importantly, the inferred silicate weathering intensity is synchronous with the strengthening of summer monsoon during the deglaciation to mid-Holocene and these changes closely follow changes in regional and global climate. Nd isotope records suggest that over the last 54 ka, detrital sediments are primarily sourced from the Myanmar rivers Salween, Sittang along with Irrawaddy. The glacial contrasts between Nd isotopic composition in the Bay of Bengal and the Andaman Sea suggest that the Andaman Sea was isolated from Bay of Bengal during low sea-levels.

## Methods

For this study, sediment samples spanning the last 54 ka of a deep-sea core (SK-168/GC-01, 11°42′N, 94°29′E; 2064 m water depth) from the Andaman Sea and four Marthaban shelf sediments off Myanmar river mouths and Arakan coast were analyzed (Fig. 1). The core SK168/GC01 comprises of three distinct sediment layers, dark yellowish brown colored clays in the top 10 cm and olive gray sediments between 30 cm to 420 cm (bottom of the core), while the 20 cm section of light olive gray sediments is sandwiched between these two layers. These sediments are extremely sticky and dominantly clayey in texture, containing tests of foraminifera. The downcore brown to grey color transition is typical of hemipelagic sediments with an oxidized top. Age model for core SK168 is well constrained^45^ which is based on five AMS ^14^C dates performed on planktic foraminiferal tests and further refined by correlating δ^18^O Globigerinoides ruber record with the low-latitude isostack curve of Martinson *et al*.^46^. The sedimentation rate deduced from this age model averages about 7.8 cm/ka with a similar rate during Holocene and between 8 and 10 cm/ka during MIS 2 and 3. Of the four shelf surface sediments sampled close to the river mouth, three are from the river mouths of Irrawaddy (Sk175/38), Salween (175/48) and Sittang (SK175/52) rivers. One sample originates from a more remote location off the Arakan Coast (SK175/3). The Arakan and Irrawaddy shelf sediments are olive green and black clays while the Salween and Sittang shelf samples are brown colored terrigenous clays. The texture of sediments is silty clay except the Irrawaddy sediment which has a sandy clay texture. All the four sediments are poor in carbonate content^47^. The sediments studied here were collected during 168^th^ and 175^th^ expeditions of *O.R.V. Sagar Kanya.*

### Sr and Nd isotope analysis of lithogenic sediments

Isotopic compositions of ^87^Sr/^86^Sr and ^143^Nd/^144^Nd were measured on the carbonate–free and Fe-Mn oxide free lithogenic fraction of the bulk sediments following a sequential leaching procedure^48^,^49^, in which carbonate was first removed using buffered acetic acid followed by Fe-Mn oxide coatings by strong reductive leaching with 1 M Hydroxylamine Hydrochloride (HH) in 25% acetic acid.

Briefly, an aliquot of ~300 mg of dry bulk sediment samples were leached in 20 ml 0.44 M acetic acid (buffered to pH5 by sodium acetate) in acid cleaned 50 ml centrifuge tubes on a shaker for 3 hrs at room temperature. The samples were then centrifuged and the acid containing dissolved carbonate was discarded. This step was repeated five times with fresh addition of buffered glacial acetic acid each time until no bubbles were discerned. The solid residue left over was washed incrementally three times with Milli-Q water, before the Fe-Mn oxides were removed by strong reductive leaching with 10 ml of 1 M HH in 25% acetic acid on a hotplate set at 90 °C for three hours. Samples were centrifuged and the residue was then rinsed four times with Milli-Q water, dried in an oven at 100 °C for a day and reweighed.

Sample dissolution and column chemistry for Sr and Nd generally follows the established procedure in our lab for the TIMS measurements^50^,^51^, but with some modifications such as dissolution in PARR Vessels and chemical separation using Savillex™ Teflon columns to accommodate Hf chemistry^52^. Approximately 100 mg of the detrital material was totally dissolved in a 3:1 ratio of double distilled concentrated HF: HNO_3_ in steel jacketed PARR vessels at 180 °C for 4 days. Dissolved samples were nitrated twice to expel fluorides and re-dissolved in 6 M HCl. Following this conversion to chloride salts, and subsequent take up in 2.5N HCl, the sample solutions were virtually free of precipitates and an aliquot of 70% of the sample solution was preserved for the determination of Sr, and Nd isotope compositions.

Chemical separation of Sr and rare earth elements (for Nd) generally follows the conventional ion-exchange chromatography procedures^52^ using Savillex™ Teflon columns (21 cm × 0.64 cm I.D.) charged with Bio-Rad^®^ AG50W-X8 (200–400). Sr was eluted with 2.5M HCl and REEs with 6N HCl. Separation of Nd from other REEs was performed in quartz columns (10 cm × 0.5 cm I.D.) containing Teflon powder coated with HDEHP [di (2-ethylhexyl) ortho phosphoric acid] with 0.25N HCl.

Sr and Nd isotope ratios were measured using a Nu Plasma MC-ICPMS (Nu Instruments, UK) in static multi-collection mode at National Geophysical Research Institute (NGRI), Hyderabad. Analyses used ‘On Peak Zeros’ correction routine for the background blank and memory. Nd was measured in dry plasma mode using a Nu DSN 100 desolvating system and Sr analysis employed wet plasma mode. Sample solutions of Sr, and Nd were prepared in 2% (v/v) optima HNO_3_. Mass fractionation for Sr and Nd isotope ratios was corrected by normalization to ^86^Sr/^88^Sr = 0.1194 and ^146^Nd/^144^Nd = 0.7219. In the course of the five analytical sessions, standards measured every fourth sample gave the following mean values: ^87^Sr/^86^Sr = 0.710254 ± 41 (58 ppm, 2σ, n = 18) for NIST SRM987 and ^143^Nd/^144^Nd = 0.512105 ± 17 (33 ppm, 2σ, n = 18) for JNdi-1. The in-run precision quantified by twice the standard error (2SE) was on an average considerably smaller (21 ppm for Sr and 24 ppm for Nd) than the external reproducibility. Measured values were normalized to the accepted ratio of 0.710245 for NIST SRM987 ^87^Sr/^86^Sr and 0.512115 for the JNdi-1 ^143^Nd/^144^Nd^53^. Epsilon Nd values were calculated using chondritic value of ^143^Nd/^144^Nd = 0.512638^54^.

Five analyses of USGS rock standard BCR-2 were used to assess the precision of the column chemistry and analytical procedure yielded ^87^Sr/^86^Sr = 0.704985 ± 38 (54 ppm, 2σ, n = 5) and ^143^Nd/^144^Nd = 0.512633 ± 7 (14 ppm, 2σ), similar to within errors of the published values of the standard^55^,^56^. All chemical procedures of leaching, digestions, chromatographic separations and purifications were performed in over pressurized HEPA filtered laminar flow hoods in the clean laboratory at NGRI, Hyderabad. The total procedural blanks were <100 pg for Sr, and ~25 pg for Nd (n = 5) insignificant compared to the size of the samples analyzed.

### Elemental Analysis

Concentrations of major, trace and REE in the lithogenic fraction of the sediments were evaluated on 1N Hydrochloric (HCl) acid treated residue after removing the authigenic, biogenic and weakly bound exchangeable components. About 2 g salt free, oven dried powdered samples were treated initially with 20 ml 1N HCl for 4 hrs, then with additional 15 ml and agitated occasionally for every 30 minutes. Once the reactions ceased, the residues were washed thoroughly with MilliQ water four times, centrifuged and dried in an oven at 60 °C.

For the measurement of major elements, 0.55 gm of the sample powder was mixed with Spectromelt^®^ A12 (Merck) flux and borate beads were prepared in a Minifuse2 induction furnace. Major elements were measured on Wavelength-Dispersive X-ray Fluorescence (XRF-WD; Axios, PANanalytical, The Netherlands) at the National Institute of Oceanography (NIO), Goa. Geological Survey of Japan reference material (JR-1) was analyzed simultaneously to monitor data quality. Accuracy and precision of the data are better than ± 4%. As the samples used were salt and carbonate-free, Na and Ca values presented here represent those present in lithogenic fraction only and thus suitable for calculating the weathering index CIA.

For trace and REE analyses, 50 mg of powdered samples were weighed into Teflon beakers and dissolved using a suprapure acid mixture of HF, HNO_3_ and HClO_4_ in a ratio of 7:3:1 on a hot plate. Following which, the digest was dissolved in 4 ml of 1:1 HNO_3_ acid and made to 100 ml final volume with Milli-Q water. Elemental concentrations were analyzed by Inductively Coupled Plasma Mass Spectrometer (ICP-MS, X-series2 of Thermo Fisher) at NIO, Goa, with Rh as internal standard^57^. Along with the samples, geochemical reference standards of MAG-1, SCo-1, and AGV-1 were analyzed and the accuracy of trace and REE with reference to these standards was better than ± 3%.

### Size analysis

Salt free sediments were wet sieved to collect the fraction less than 63 μm (silt and clay). Biogenic components (i.e. carbonate, organic matter and opal) in this fraction were removed sequentially by treatment with 1:4 acetic acid, 3% H_2_O_2_ and hot Na_2_CO_3._ The grain size distribution of the treated sediments were measured using Malvern laser particle size analyzer (Mastersizer, 2000) with a Hydro 2000 MU wet sampling accessory using procedures^58^ at NIO, Goa. Accuracy and analytical precision for D_50_ (median gain size) was better than ± 1%.

### Geochemical and isotopic proxies of weathering

Geochemical discrimination parameters and proxies of weathering such as the chemical index of alteration (CIA), elemental Al/K, Rb/Sr and ^87^Sr/^86^Sr isotope ratios of the detrital sediments are used here to evaluate the intensity of chemical weathering the sediments have undergone in the source regions. The sensitivity of these proxies to track and quantify the chemical weathering intensity is well established^59^,^60^,^61^,^62^,^63^,^64^, but is only limited by grain size effect if any due to hydrodynamic sorting of sediments. CIA represents the molar proportions of more labile Na, K and Ca compared to immobile Al. A CIA value of 100 indicates intense chemical weathering, whereas values of 45–55 indicate virtually no weathering. Al is relatively conservative during weathering process, while K tends to be enriched in weathering products during moderate weathering, but depleted during extreme weathering. Therefore, high Al/K ratio in sediments can be regarded as to extreme chemical weathering (e.g., Nesbitt *et al*.^65^). Variation in Rb/Sr and ^87^Sr/^86^Sr ratios primarily reflects the geochemistry of fluid-rock interaction, where more mobile Sr will be leached out partly into solution during weathering, while the immobile Rb stays with the residue. Thus during periods of intense chemical weathering, leaching of more Sr into solution could lower Rb/Sr and ^87^Sr/^86^Sr ratios and vice versa if the physical weathering dominates.

### Oceanography of the Andaman Sea

The present day oceanography of the Andaman Sea has been described by Sijinkumar *et al*.,^66^ (and references therein). Andaman Sea is a marginal sea located in the eastern part of the north-eastern Indian Ocean (Fig. 1). As in the case of the northern Bay of Bengal, the Andaman Sea receives annually large amount of fresh water from the Irrawaddy catchment with most of the outflow occurring during the summer to late fall^29^. As a result of a fresh water influx, salinity reduces to a minimum value during summer monsoon (July to August^9^). The annual average salinity at our core location (SK 168) is nearly 31.5‰^66^. As a result, a larger salinity gradient exists between the peak and inter monsoon periods. The annual surface water temperature ranges from 28 to 30 °C and is well mixed to a depth of 50 m leading to stratification which hinders vertical mixing^10^. The Andaman Sea experiences, a seasonal reversal in surface circulation similar to that of the Arabian Sea. The maximum water depth in the Andaman Sea is 4400 m and it is inter-connected with the BoB via several openings viz., the Deep Prepares Channel, Ten Degree Channel, and the Great Channel. Similar to Arabian Sea, upwelling induced productivity changes is also reported from the Andaman Sea with a lower intensity mainly driven by cyclonic eddies. Biological productivity in the offshore Andaman region is ~0.8–1.0 mg C/m^2^/d compared to lesser values (<0.6 mg C/m^2^/d) observed in the coastal areas^67^.

### Data availability statement

The entire geochemical and isotopic data related to the paper is made available in Tables S1 and S2 in the supplementary information.

## Additional Information

**How to cite this article:** Miriyala, P. *et al*. Increased chemical weathering during the deglacial to mid-Holocene summer monsoon intensification. *Sci. Rep.* **7**, 44310; doi: 10.1038/srep44310 (2017).

**Publisher's note:** Springer Nature remains neutral with regard to jurisdictional claims in published maps and institutional affiliations.

## Supplementary Material

Supplementary Information
