# Supplementary material for: Increased chemical weathering during the deglacial to mid-Holocene summer monsoon intensification
Source: Sci Rep. 2017 Mar 17;7:44310. doi: 10.1038/srep44310 (PMC5355878; doi:10.1038/srep44310)
Supplement: Supplementary Information [file srep44310-s1.doc]

**Supplementary information for MS#SREP-16-38461**

**Increased chemical weathering during the deglacial to mid-Holocene summer monsoon intensification.**

Pavan Miriyala, Sukumaran N.P, Nagender Nath B, Ramamurty P.B, Sijinkumar A.V, Vijayagopal B, Ramaswamy V and Tyson Sebastian

Tables

**Table S1** Nd and Sr isotopic composition of the SK168/GC01 in the Andaman Sea and Myanmar shelf sediments.

Table S2 Major and trace element content and calculated CIA of the SK168/GC01 in the Andaman Sea and Myanmar shelf sediments.

Figures

**Figure S3**. Triangular plots of a) A-CN-K (Al_2_O_3_-CaO+Na_2_O-K_2_O); b) A-CNK-FM (Al_2_O_3_ - CaO+Na_2_O+K_2_O - FeO+MgO); c) La-Th-Sc of SK168 and shelf (river mouth) sediments along with average composition of UCC-Upper Continental Crust^1^, tonalite, granodiorite, granite, basalt and ophiolite.

**Figure S4**. ε_Nd_ versus ^87^Sr/^86^Sr of present work and published values for Andaman sediments. Also plotted are composition of diorite, andesite from Indo-Burman ranges^2^, ophiolites of Andaman Islands, ash layers in SK234 (western Andaman Sea) and Barren Island rocks^3^.

**Figure S5.** Present day and reconstructed river catchments during interglacial and glacial periods respectively. Increased catchments during interglacials due to increased vegetated and soil zone areas. Additional catchments (orange) during interglacials would facilitate produce more weathered material. During glacials, due to less catchment area, low infiltration and high runoff are expected. Exposed shelf during glacials (reconstructed - shaded area, below 120MSL of present day). This map is generated by using ArcGIS programme, E.S.R.I. "10.1."*Redlands, California: ESRI* (2012).

**References:**

1. Taylor, S R, and McLennan, S. M. *The continental crust: its composition and evolution*. (1985).

2. Mitchell, A., Chung, S.-L., Oo, T., Lin, T.-H. & Hung, C.-H. Zircon U–Pb ages in Myanmar: Magmatic–metamorphic events and the closure of a neo-Tethys ocean? *J. Asian Earth Sci.* **56,** 1–23 (2012).

3. Awasthi, N., Ray, J.S., Singh, A.K., Band, S.T., Rai, V. K. Provenance of the Late Quaternary sediments in the Andaman Sea: Implications for monsoon variability and ocen circulation. *Geochemistry Geophys. Geosystems* **15,** 3890–3906 (2014).

4. Jacobsen, S. B. & Wasserburg, G. J. Sm-Nd isotopic evolution of chondrites. *Earth Planet. Sci. Lett.* **50,** 139–155 (1980).

5. DePaolo, D. J. Crustal growth and mantle evolution: inferences from models of element transport and Nd and Sr isotopes. *Geochim. Cosmochim. Acta* **44,** 1185–1196 (1980).

**Tables**

***Table S1: Nd and Sr isotopic composition of the SK168/GC01 in the Andaman Sea and Myanmar shelf sediments***

|  | | | | | | |  |
| --- | --- | --- | --- | --- | --- | --- | --- |
| **Mean Depth (cm)** | **Age cal ka BP** | **^87^Sr/^86^Sr** | **±2SE** | **^143^Nd/^144^Nd** | **±2SE** | **ε_Nd_**  **±0.33** | **T_DM_ Ga** |
| 1 | 0.88 | 0.717078 | 16 | 0.512137 | 6 | -9.76 | 1.20 |
| 17 | 2.90 | 0.719591 | 16 | 0.512131 | 6 | -9.90 | 1.14 |
| 25 | 3.92 | 0.718396 | 14 | 0.512119 | 5 | -10.13 | 1.41 |
| 33 | 4.93 | 0.720297 | 11 | 0.512112 | 6 | -10.25 | 1.34 |
| 37 | 5.43 | 0.719583 | 18 | 0.512138 | 6 | -9.75 | 1.29 |
| 41 | 5.94 | 0.719562 | 11 | 0.512105 | 6 | -10.39 | 1.46 |
| 49 | 6.95 | 0.717136 | 17 | 0.512157 | 6 | -9.38 | 1.39 |
| 56 | 7.83 | 0.717396 | 15 | 0.512125 | 4 | -10.00 | 1.46 |
| 61 | 8.47 | 0.718760 | 11 | 0.512135 | 13 | -9.82 | 1.33 |
| 66 | 9.10 | 0.719178 | 20 | 0.512163 | 5 | -9.27 | 1.38 |
| 71 | 9.73 | 0.718911 | 20 | 0.512156 | 6 | -9.40 | 1.34 |
| 73.5 | 10.05 | 0.719118 | 11 | 0.512153 | 6 | -9.46 | 1.37 |
| 77.5 | 10.50 | 0.719277 | 15 | 0.512164 | 7 | -9.24 | 1.37 |
| 83.5 | 11.10 | 0.718887 | 18 | 0.512164 | 7 | -9.25 | 1.33 |
| 88.5 | 11.59 | 0.719131 | 16 | 0.512153 | 5 | -9.45 | 1.29 |
| 106 | 13.60 | 0.720016 | 10 | 0.512139 | 5 | -9.73 | 1.19 |
| 116 | 14.87 | 0.721100 | 14 | 0.512118 | 7 | -10.15 | 1.39 |
| 126 | 16.14 | 0.722263 | 21 | 0.512108 | 4 | -10.33 | 1.35 |
| 131 | 16.78 | 0.721732 | 10 | 0.512109 | 7 | -10.31 | 1.30 |
| 138.5 | 17.74 | 0.715421 | 11 | 0.512129 | 4 | -9.92 | 1.32 |
| 148.5 | 19.01 | 0.721096 | 11 | 0.512115 | 6 | -10.21 | 1.30 |
| 156 | 19.97 | 0.722400 | 30 | 0.512108 | 6 | -10.34 | 1.33 |
| 161 | 20.61 | 0.722180 | 17 | 0.512111 | 6 | -10.27 | 1.33 |
| 168.5 | 21.56 | 0.722484 | 11 | 0.512111 | 8 | -10.28 | 1.32 |
| 171 | 21.88 | 0.722560 | 13 | 0.512111 | 8 | -10.28 | 1.34 |
| 178.5 | 22.84 | 0.722405 | 18 | 0.512105 | 6 | -10.39 | 1.35 |
| 186 | 23.79 | 0.718853 | 23 | 0.512093 | 4 | -10.62 | 1.26 |
| 188.5 | 24.11 | 0.723022 | 29 | 0.512101 | 5 | -10.47 | 1.45 |
| 191 | 24.79 | 0.723131 | 10 | 0.512084 | 5 | -10.81 | 1.39 |
| 198.5 | 26.85 | 0.723799 | 25 | 0.512062 | 6 | -11.24 | 1.43 |
| 202.5 | 27.95 | 0.723408 | 11 | 0.512091 | 6 | -10.67 | 1.34 |
| 227.5 | 33.13 | 0.722420 | 11 | 0.512092 | 4 | -10.64 | 1.35 |
| 247.5 | 35.27 | 0.722541 | 9 | 0.512100 | 4 | -10.49 | 1.31 |
| 252.5 | 35.80 | 0.722846 | 10 | 0.512109 | 6 | -10.31 | 1.30 |
| 282.5 | 39.01 | 0.722574 | 17 | 0.512095 | 3 | -10.59 | 1.33 |
| 312.5 | 42.22 | 0.723104 | 25 | 0.512088 | 3 | -10.73 | 1.37 |
| 322.5 | 43.29 | 0.723043 | 12 | 0.512104 | 7 | -10.41 | 1.37 |
| 332.5 | 44.36 | 0.722081 | 20 | 0.512110 | 5 | -10.29 | 1.26 |
| 352.5 | 46.50 | 0.723055 | 15 | 0.512123 | 5 | -10.05 | 1.34 |
| 357.5 | 47.04 | 0.723244 | 16 | 0.512125 | 5 | -10.01 | 1.30 |
| 377.5 | 49.18 | 0.723361 | 15 | 0.512118 | 5 | -10.14 | 1.30 |
| 387.5 | 50.25 | 0.723228 | 30 | 0.512108 | 5 | -10.33 | 1.36 |
| 407.5 | 52.39 | 0.724377 | 13 | 0.512089 | 4 | -10.71 | 1.37 |
| 417.5 | 53.46 | 0.722935 | 18 | 0.512100 | 6 | -10.49 | 1.28 |
|  |  |  |  |  |  |  |  |
| **Shelf samples off river mouths of Myanmar and Arakan coast** | | | | |  |  |  |
| SK175/3 | Arakan | 0.714849 | 14 | 0.512183 | 8 | -8.88 | 1.26 |
| SK175/38 | Irrawaddy | 0.713429 | 11 | 0.512191 | 8 | -8.72 | 1.28 |
| SK175/48 | Sittang | 0.720063 | 14 | 0.512065 | 7 | -11.19 | 1.44 |
| SK175/52 | Salween | 0.721543 | 14 | 0.512106 | 5 | -10.37 | 1.36 |

2SE (twice the standard errors) are on the final quoted significant figures. Calculated with (^143^Nd/^144^Nd)_CHUR_ = 0.512638^4^ . Errors for ε_Nd_ are given as 2SD, the external reproducibility of JNdi ^143^Nd/^144^Nd =0.512105 ± 17 which translates to ±0.33 ε_Nd_ units. (TDM) Depleted mantle model ages estimate using measured ^143^Nd/^144^Nd and ^147^Sm/^144^Nd ratios following DePaolo (1980)^5^.

| *Table S2: Major and trace element content and calculated CIA of the SK168/GC01 in the Andaman Sea and Myanmar shelf sediments* | | | | | | | | | | | | |
| --- | --- | --- | --- | --- | --- | --- | --- | --- | --- | --- | --- | --- |
| **Mean depth (cm)** | **Age cal**  **ka BP** | **Al_2_O_3_** | **CaO** | **Na_2_O** | **K_2_O** | **Rb** | **Sr** | **Sm** | **Nd** | **Al/K** | **Rb/Sr** | **CIA*** |
|  |  | % | % | % | % | ppm | ppm | ppm | ppm |  |  |  |
| 1 | 0.88 | 17.1 | 1.5 | 0.9 | 2.3 | 124 | 102 | 2.9 | 17.7 | 4.8 | 1.2 | 81.6 |
| 5 | 1.39 | 17.3 | 1.3 | 0.9 | 2.3 | 130 | 120 | 2.8 | 15.2 | 4.7 | 1.1 | 80.9 |
| 9 | 1.89 | 17.3 | 1.2 | 0.9 | 2.4 | 134 | 128 | 3.9 | 20.2 | 4.7 | 1.0 | 81.3 |
| 17 | 2.90 | 17.4 | 1.1 | 0.8 | 2.3 | 137 | 87 | 2.4 | 16.0 | 4.8 | 1.6 | 82.2 |
| 21 | 3.41 | 17.2 | 1.3 | 0.9 | 2.3 | 134 | 129 | 3.8 | 19.9 | 4.8 | 1.0 | 81.3 |
| 25 | 3.92 | 17.1 | 1.2 | 0.9 | 2.4 | 127 | 118 | 3.5 | 18.6 | 4.6 | 1.1 | 81.1 |
| 33 | 4.93 | 17.6 | 0.8 | 0.6 | 2.4 | 149 | 95 | 3.9 | 21.7 | 4.7 | 1.6 | 82.8 |
| 37 | 5.43 | 17.5 | 1.1 | 0.7 | 2.4 | 140 | 103 | 3.5 | 20.0 | 4.7 | 1.4 | 82.4 |
| 41 | 5.94 | 17.6 | 1.1 | 0.8 | 2.4 | 135 | 124 | 3.5 | 18.3 | 4.6 | 1.1 | 81.6 |
| 49 | 6.95 | 17.9 | 1.2 | 0.8 | 2.3 | 124 | 111 | 3.4 | 17.5 | 4.9 | 1.1 | 82.1 |
| 56 | 7.83 | 17.3 | 1.3 | 0.9 | 2.3 | 131 | 125 | 3.5 | 18.2 | 4.8 | 1.0 | 81.2 |
| 61 | 8.47 | 18.0 | 0.7 | 0.7 | 2.4 | 140 | 89 | 3.5 | 19.4 | 4.9 | 1.6 | 83.1 |
| 66 | 9.10 | 17.7 | 0.9 | 0.8 | 2.4 | 144 | 129 | 3.7 | 19.3 | 4.7 | 1.1 | 82.0 |
| 71 | 9.73 | 17.6 | 1.1 | 0.8 | 2.4 | 136 | 128 | 3.5 | 18.9 | 4.7 | 1.1 | 81.6 |
| 73.5 | 10.05 | 17.5 | 0.9 | 0.7 | 2.3 | 141 | 129 | 4.0 | 21.1 | 4.8 | 1.1 | 82.3 |
| 77.5 | 10.50 | 17.7 | 1.3 | 0.8 | 2.4 | 144 | 132 | 3.7 | 19.6 | 4.7 | 1.1 | 82.1 |
| 83.5 | 11.10 | 17.8 | 1.2 | 0.8 | 2.4 | 147 | 128 | 3.5 | 18.7 | 4.8 | 1.1 | 82.1 |
| 88.5 | 11.59 | 18.8 | 1.0 | 0.8 | 2.5 | 138 | 111 | 2.6 | 14.6 | 4.8 | 1.2 | 82.6 |
| 101 | 12.96 | 18.0 | 1.3 | 0.7 | 2.4 | 134 | 91 | 1.9 | 12.6 | 4.7 | 1.5 | 82.5 |
| 106 | 13.60 | 18.2 | 1.0 | 0.9 | 2.8 | 156 | 96 | 2.6 | 16.2 | 4.1 | 1.6 | 80.2 |
| 111 | 14.23 | 18.1 | 1.1 | 0.7 | 2.5 | 141 | 109 | 1.9 | 11.5 | 4.6 | 1.3 | 82.2 |
| 116 | 14.87 | 18.4 | 0.8 | 0.7 | 2.6 | 140 | 111 | 3.6 | 19.5 | 4.6 | 1.3 | 82.6 |
| 121 | 15.51 | 18.7 | 0.8 | 0.7 | 2.6 | 142 | 95 | 2.0 | 12.0 | 4.5 | 1.5 | 82.2 |
| 126 | 16.14 | 18.7 | 1.1 | 0.8 | 2.7 | 137 | 105 | 3.2 | 17.9 | 4.4 | 1.3 | 81.7 |
| 131 | 16.78 | 18.6 | 1.2 | 0.8 | 2.7 | 142 | 108 | 2.9 | 17.1 | 4.4 | 1.3 | 81.6 |
| 138.5 | 17.74 | 18.6 | 1.9 | 0.9 | 2.6 | 139 | 114 | 3.5 | 19.4 | 4.6 | 1.2 | 81.1 |
| 141 | 18.06 | 18.4 | 1.8 | 0.9 | 2.6 | 135 | 111 | 3.3 | 18.5 | 4.6 | 1.2 | 81.0 |
| 148.5 | 19.01 | 18.5 | 1.0 | 0.8 | 2.8 | 150 | 95 | 3.2 | 18.7 | 4.3 | 1.6 | 81.0 |
| 151 | 19.33 | 18.3 | 1.2 | 0.9 | 2.7 | 147 | 101 | 3.0 | 17.5 | 4.3 | 1.4 | 80.6 |
| 156 | 19.97 | 18.3 | 1.0 | 0.8 | 2.7 | 160 | 106 | 3.6 | 20.2 | 4.2 | 1.5 | 81.0 |
| 161 | 20.61 | 18.5 | 1.0 | 0.8 | 2.8 | 159 | 106 | 3.8 | 21.5 | 4.2 | 1.5 | 80.9 |
| 163.5 | 20.92 | 18.3 | 0.9 | 0.8 | 2.8 | 160 | 100 | 3.7 | 21.1 | 4.2 | 1.6 | 81.0 |
| 168.5 | 21.56 | 18.3 | 1.1 | 0.9 | 2.8 | 154 | 104 | 3.0 | 17.3 | 4.2 | 1.5 | 80.1 |
| 171 | 21.88 | 18.0 | 0.8 | 0.8 | 2.8 | 166 | 107 | 3.8 | 21.6 | 4.2 | 1.5 | 80.5 |
| 178.5 | 22.84 | 18.2 | 0.8 | 0.8 | 2.8 | 164 | 100 | 2.6 | 16.0 | 4.1 | 1.7 | 80.6 |
| 181 | 23.15 | 18.1 | 1.1 | 0.7 | 2.7 | 147 | 85 | 2.0 | 13.6 | 4.2 | 1.7 | 81.1 |
| 186 | 23.79 | 18.0 | 1.1 | 0.8 | 2.5 | 138 | 122 | 3.5 | 18.4 | 4.6 | 1.1 | 82.0 |
| 188.5 | 24.11 | 18.0 | 1.1 | 0.8 | 2.8 | 165 | 105 | 2.9 | 17.5 | 4.1 | 1.6 | 80.3 |
| 191 | 24.79 | 18.4 | 0.7 | 0.8 | 2.9 | 160 | 92 | 3.5 | 19.4 | 4.1 | 1.7 | 80.9 |
| 196 | 26.16 | 18.3 | 1.2 | 0.7 | 2.9 | 168 | 110 | 3.8 | 21.5 | 4.1 | 1.5 | 81.1 |
| 198.5 | 26.85 | 18.4 | 0.8 | 0.9 | 3.0 | 162 | 99 | 3.9 | 21.6 | 4.0 | 1.6 | 79.9 |
| 202.5 | 27.95 | 18.4 | 1.0 | 0.8 | 2.8 | 170 | 105 | 3.9 | 22.1 | 4.1 | 1.6 | 80.7 |
| 212.5 | 30.69 | 18.3 | 0.8 | 0.8 | 2.8 | 173 | 103 | 3.8 | 21.1 | 4.1 | 1.7 | 80.7 |
| 227.5 | 33.13 | 18.5 | 0.8 | 0.8 | 2.8 | 165 | 100 | 3.6 | 20.6 | 4.2 | 1.7 | 80.8 |
| 237.5 | 34.20 | 18.5 | 1.2 | 1.0 | 2.8 | 154 | 88 | 2.1 | 13.5 | 4.3 | 1.7 | 80.2 |
| 247.5 | 35.27 | 17.7 | 1.3 | 0.7 | 2.7 | 165 | 106 | 3.3 | 19.3 | 4.2 | 1.6 | 81.3 |
| 257.5 | 36.34 | 18.8 | 0.8 | 0.8 | 2.8 | 175 | 106 | 4.0 | 22.6 | 4.3 | 1.7 | 81.1 |
| 277.5 | 38.48 | 18.5 | 0.8 | 0.7 | 2.8 | 167 | 89 | 2.5 | 16.4 | 4.3 | 1.9 | 81.9 |
| 282.5 | 39.01 | 18.8 | 0.7 | 0.7 | 2.8 | 177 | 96 | 3.8 | 21.5 | 4.2 | 1.8 | 81.8 |
| 292.5 | 40.08 | 18.5 | 0.7 | 0.7 | 2.9 | 181 | 98 | 4.1 | 22.9 | 4.1 | 1.8 | 81.4 |
| 312.5 | 42.22 | 18.5 | 0.6 | 0.7 | 2.9 | 181 | 97 | 3.7 | 21.4 | 4.1 | 1.9 | 81.1 |
| 322.5 | 43.29 | 18.4 | 0.7 | 0.7 | 2.9 | 178 | 98 | 3.7 | 20.4 | 4.1 | 1.8 | 81.1 |
| 332.5 | 44.36 | 18.6 | 0.6 | 0.7 | 2.9 | 178 | 89 | 3.0 | 17.8 | 4.1 | 2.0 | 81.5 |
| 342.5 | 45.43 | 18.7 | 0.5 | 0.7 | 2.9 | 184 | 92 | 3.2 | 18.6 | 4.1 | 2.0 | 81.3 |
| 352.5 | 46.50 | 18.7 | 0.8 | 0.7 | 2.9 | 178 | 97 | 3.7 | 20.8 | 4.1 | 1.8 | 81.3 |
| 357.5 | 47.04 | 18.6 | 0.7 | 0.7 | 2.9 | 157 | 85 | 2.9 | 16.7 | 4.1 | 1.9 | 81.3 |
| 367.5 | 48.11 | 18.4 | 0.7 | 0.8 | 2.9 | 173 | 87 | 2.3 | 14.7 | 4.0 | 2.0 | 80.6 |
| 377.5 | 49.18 | 18.1 | 0.9 | 0.7 | 2.9 | 170 | 104 | 3.8 | 21.9 | 4.0 | 1.6 | 80.8 |
| 387.5 | 50.25 | 18.5 | 0.6 | 0.7 | 2.8 | 172 | 91 | 3.4 | 18.7 | 4.2 | 1.9 | 81.6 |
| 397.5 | 51.32 | 18.4 | 0.8 | 0.7 | 2.9 | 172 | 93 | 3.5 | 19.3 | 4.1 | 1.8 | 81.0 |
| 407.5 | 52.39 | 18.3 | 0.8 | 0.7 | 3.0 | 179 | 97 | 3.8 | 21.1 | 3.9 | 1.8 | 80.4 |
| 417.5 | 53.46 | 18.5 | 0.6 | 0.7 | 2.9 | 182 | 89 | 2.8 | 17.0 | 4.0 | 2.1 | 81.2 |
|  |  |  |  |  |  |  |  |  |  |  |  |  |
| ***Shelf samples off river mouths of Myanmar and Arakan coast*** | | | | | | | | | | | | |
| SK175/3 | Arakan | 15.3 | 0.3 | 0.9 | 1.9 | 90 | 62 | 3.6 | 19.8 | 5.0 | 1.5 | 81.3 |
| SK175/38 | Irrawaddy | 16.3 | 0.7 | 1.9 | 2.1 | 80 | 85 | 2.4 | 13.3 | 4.9 | 0.9 | 75.3 |
| SK175/48 | Sittang | 20.8 | 0.3 | 1.1 | 2.7 | 155 | 81 | 4.0 | 22.0 | 4.9 | 1.9 | 81.5 |
| SK175/52 | Salween | 20.5 | 0.3 | 1.1 | 2.7 | 153 | 75 | 3.9 | 21.8 | 4.8 | 2.0 | 81.3 |


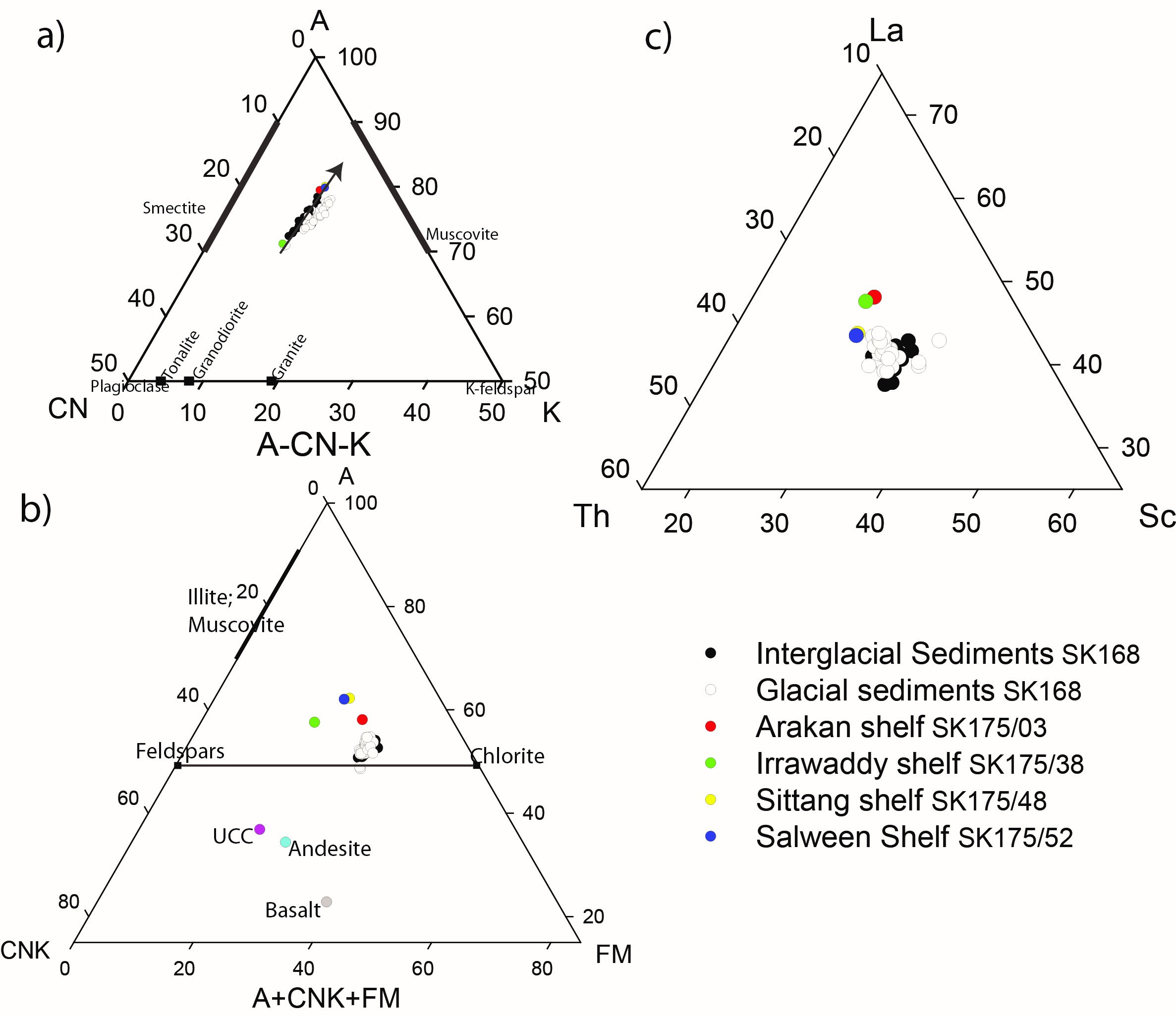


Figure S3. Triangular plots of a) A-CN-K (Al_2_O_3_-CaO+Na_2_O-K_2_O); b) A-CNK-FM (Al_2_O_3_ - CaO+Na_2_O+K_2_O - FeO+MgO); c) La-Th-Sc of SK168 and shelf (river mouth) sediments along with average composition of UCC-Upper Continental Crust^1^, tonalite, granodiorite, granite, basalt and ophiolite.


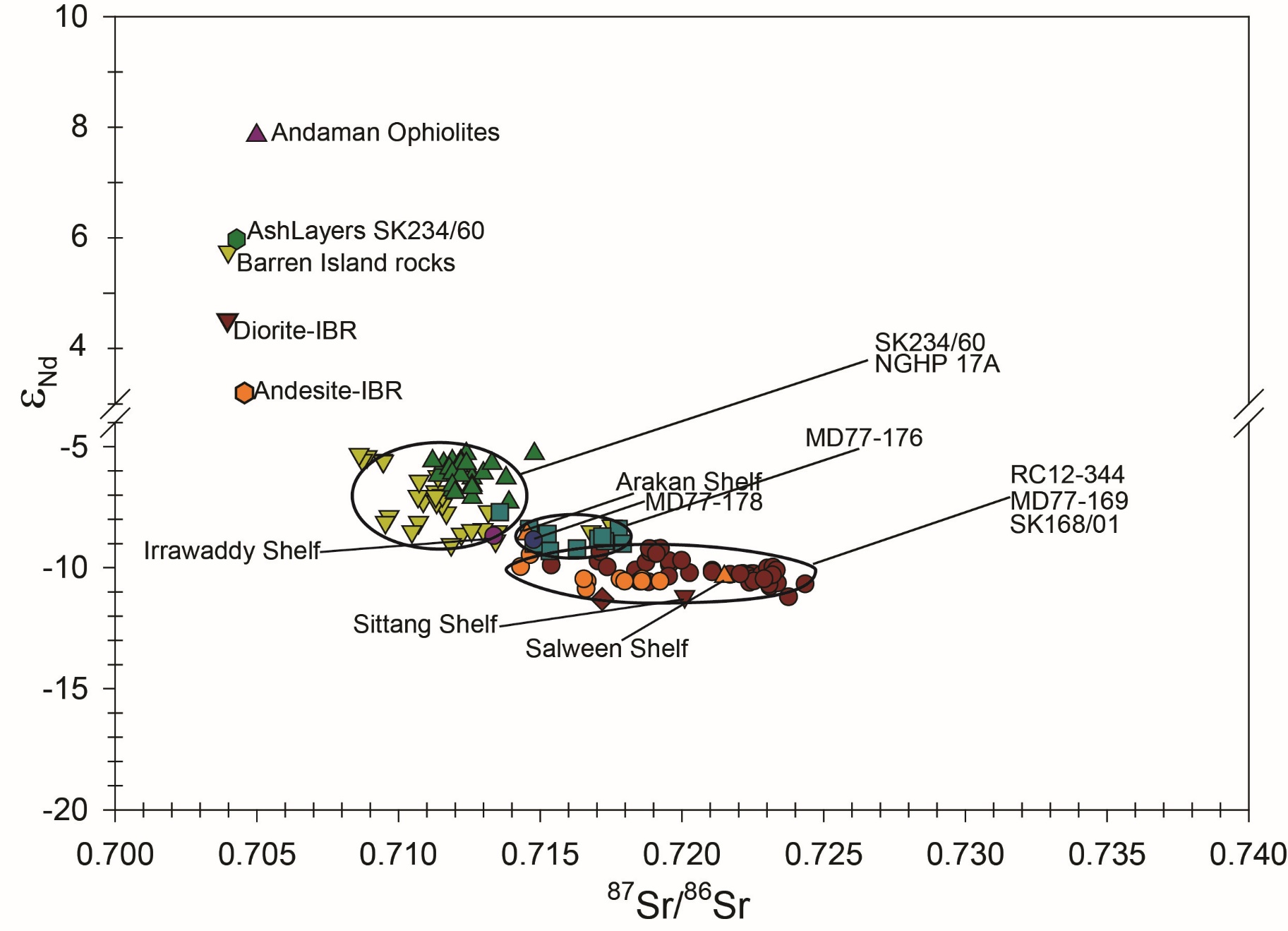


Figure S4. ε_Nd_ versus ^87^Sr/^86^Sr of present work and published values for Andaman sediments. Also plotted are composition of diorite, andesite from Indo-Burman ranges^2^, ophiolites of Andaman Islands, ash layers in SK234 (western Andaman Sea) and Barren Island rocks^3^.


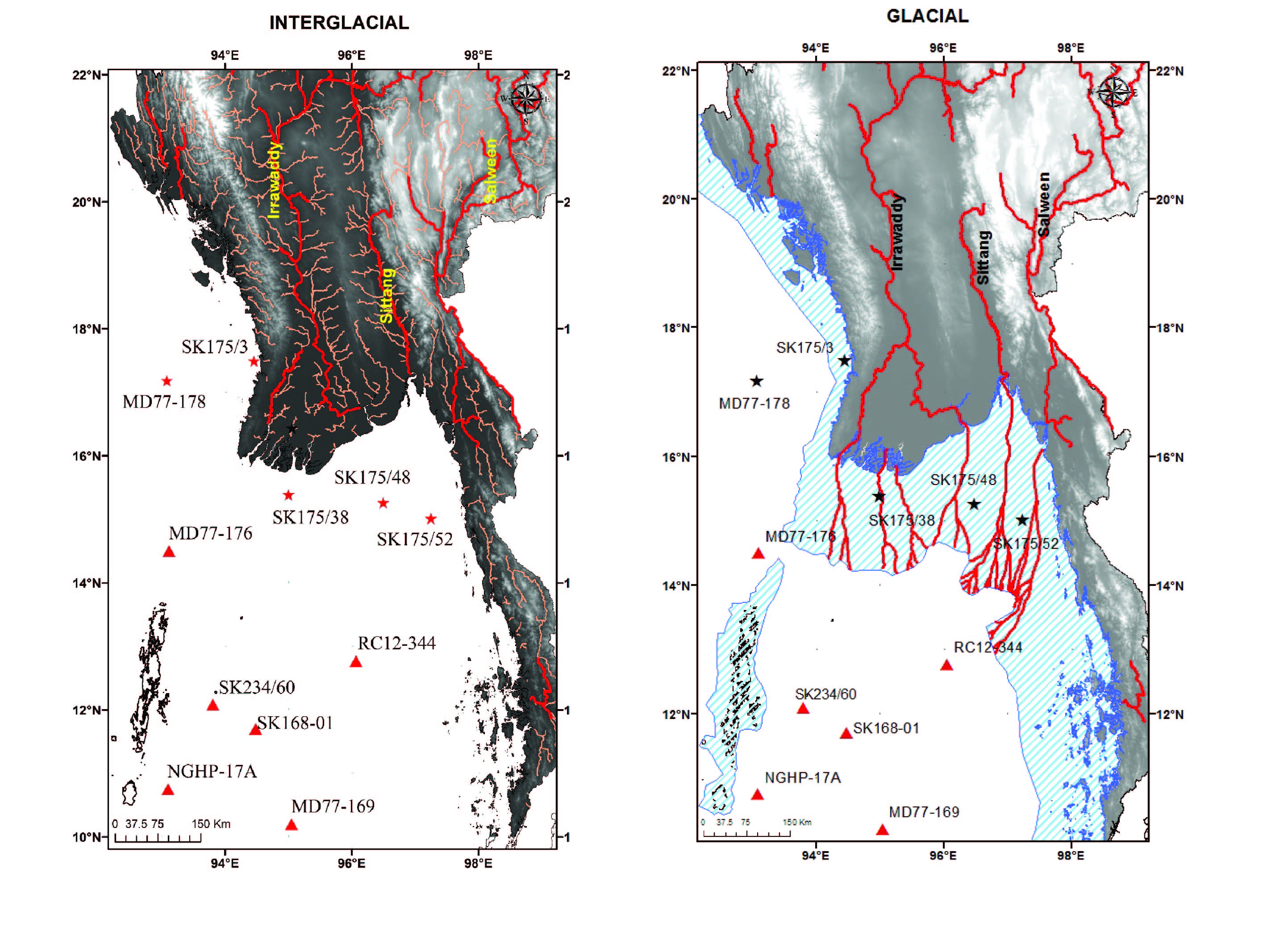


Figure S5. Present day and reconstructed river catchments during interglacial and glacial periods respectively. Increased catchments during interglacials due to increased vegetated and soil zone areas. Additional catchments (orange) during interglacials would facilitate produce more weathered material. During glacials, due to less catchment area, low infiltration and high runoff are expected. Exposed shelf during glacials (reconstructed - shaded area, below 120MSL of present day). This map is generated by using ArcGIS programme, E.S.R.I. "10.1."*Redlands, California: ESRI* (2012).
